# Domestic Intelligence

**Published:** 1800-03

**Authors:** 


### *Domeſtic Intelligence.*

The Surgeons of the Mancheſter Infirmary, having heard with ſatisfaction of the Society lately inſtituted in London for the Vaccine Inoculation, which is proved by experience to preſerve the conſtitution of thoſe who have ſubmitted to it, againſt the contagion of the Small Pox; and being convinced that much advantage would be derived from a more general Inoculation of the Cow Pox, propoſe to extend the benefits of the diſcovery to the objects of that Charity. They ſtate, by public advertiſement, their intention of practiſing the Vaccine Inoculation the enſuing ſeaſon, which will commence on the 10th day of March next; but obſerve, however, that they do not mean to relinquiſh entirely the Inoculation for the Small Pox.——The ſame advertiſement contains a reſolution of the Weekly Board of that Hoſpital, to print the Communications from Surgeons of thoſe Charities on the above ſubject, in the Mancheſter news-papers.

---

Mr. Fox will commence his next Courſe of Lectures on the Diſeaſes and Operations of the Teeth, on Monday, March 3, at half paſt Five o'clock.—The Lectures will be delivered at Mr. Cox's, St. Thomas's Street, near St. Thomas's Hoſpital.

---

Mr. John Pearson is engaged in writing a Work on the Venereal Diſeaſe, the compoſition of which is nearly finiſhed, ſo that it will be ſubmitted to the Preſs in the courſe of the enſuing month.

